# Prostate Cancer after Percutaneous Arterial Embolization of the Prostate: A Case Report

**DOI:** 10.3390/diagnostics12102378

**Published:** 2022-09-30

**Authors:** Ying-Chieh Chang, Szu-Ju Chen, Wei-Hsuan Huang, Chi-Ping Huang, Yung-Hsiang Chen, Wen-Chi Chen

**Affiliations:** 1 Department of Urology, Department of Medical Research, China Medical University Hospital, Taichung 40447, Taiwan; 2 Division of Urology, Department of Surgery, Taichung Veterans General Hospital, Taichung 40705, Taiwan; 3 Department of Urology, Everan Hospital, Taichung 41159, Taiwan; 4 School of Medicine, Graduate Institute of Integrated Medicine, College of Chinese Medicine, China Medical University, Taichung 40402, Taiwan; 5 Department of Psychology, College of Medical and Health Science, Asia University, Taichung 41354, Taiwan

**Keywords:** percutaneous arterial embolization of the prostate, Warburg effect, prostate cancer, benign prostate hyperplasia, hypoxia

## Abstract

We report a patient with prostate cancer found 2 years after percutaneous arterial embolization (PAE) of the prostate with a rapid increase in prostate specific antigen (PSA) 3 months later, even though the initial result was low. He did not consult a urologist during or after PAE until acute urinary retention developed. The clinical stage was cT2cN1M1b with Gleason grade 5 + 5 = 10. An increase in PSA a short interval after PAE may suggest the presence of prostate cancer. We suggest that patients undergoing PAE should consult a urologist, and that PSA levels should be checked every 3 months in the first year after PSA.

An 85-year-old male patient presented with a history of diabetic nephropathy and benign prostate hyperplasia (BPH) with lower urinary tract symptoms (LUTS) under oral alfa-blocker treatment. He was a non-smoker and had no family history of prostate cancer. His initial PSA level was 4.2 ng/dL, and transrectal ultrasonography showed that his prostate was 60 g in weight on 3 April 2019. He underwent PAE by a cardiologist on 18 April 2019, after an outpatient visit for hypertension. His PSA level decreased to 1.6 ng/dL one month later, but then gradually increased to 2.83 ng/dL three months later and 2.91 ng/dL five months later (29 October 2019). He underwent transrectal ultrasonography again six months postoperatively, which showed that his prostate weighed 23 g. He was then lost to follow-up until 2021. He returned to our outpatient clinic on account of dysuria, urge incontinence and perineal discomfort in March 2021. As pyuria was found in a urine routine test, he was treated with antibiotics initially. His PSA level was 15.41 in March 2021. He did not respond well to oral medications for LUTS, and he was then referred to urologic outpatient services due to acute urinary retention on May 18th. His serum PSA level was 12.58 ng/dL, and an echogram showed a prostate weight of 97 g. A digital rectal examination (DRE) revealed a firm prostate with an irregular surface, and malignancy of the prostate was highly suspected. We then performed a transrectal prostate biopsy and TURP on 29 June 2021. The intraoperative findings were a papillary lesion at the surface of necrotic tissue at the transition zone, and the total resected chips weighed 61 g. The pathology report eventually showed adenocarcinoma, Gleason score 5 + 5 = 10 (>5%). An abdominal computed tomography revealed multiple lymph node metastases at the paraaortic area (Figure 1), and a bone scan revealed boney metastasis at the L-spine (Figure 2). The clinical stage was cT2cN1M1b. He was then treated with regular hormone therapy. The last checked PSA value was 1.5 ng/dL on 15 June 2022. Written informed consent was obtained from the patient for the publication of this case report.

BPH is commonly seen in older male patients. BPH may cause bladder outlet obstruction (BOO), and therefore patients may develop a variety of symptoms related to voiding urine such as LUTS [1]. Acute urinary retention, urinary tract infection, vesicle stones and chronic kidney disease may occur if the symptoms progress. The diagnosis of BPH at a urologic clinic includes a DRE urine routine test, renal function examination, International Prostate Symptom Score (IPSS), checking prostate specific antigen (PSA) and questionnaires regarding the quality of life (QoL). Uroflow examinations include max flow rate (Qmax), average flow rate and post-voiding (PV) volume measurements. Ultrasonographic examinations can be used to evaluate a large prostate, and the presence of urolithiasis and hematuria [2].

The treatment of BPH includes medications and surgery depending on the patient’s degree of LUTS. Medical treatment includes alfa-blockers, 5-α-reductase and anticholinergic agents [3]. According to the American Urological Association (AUA) and European Urological Association (EAU) guidelines, TURP is the gold standard surgical treatment for BPH [2]. Several minimally invasive surgeries have also been introduced as alternatives to standard TURP for BPH with LUTS, including Rezūm water vapor thermal therapy, transurethral microwave therapy (TUMT), the UroLift system, transurethral needle ablation (TUNA) and percutaneous arterial embolization (PAE) [4,5].

PAE of the prostate was first reported by Carnevale et al. in 2012 with two early successful cases [6]. As an alternative to surgical treatment, PAE has the potential to improve voiding symptoms in patients with BPH. The procedure is generally performed by radiologists or cardiologists by trans-femoral or trans-radial puncture and superselective embolization of bilateral prostate arteries [7]. Initial results have been promising with few complications, especially for patients at high risk of surgery. Significant improvements of IPSS, post-voiding urine volume, Qmax and QoL were reported in a systematic review by Kuang et al. [8]. However, Kao et al. compared TURP and PAE in a randomized controlled trial and reported that the TURP group had better symptom relief in term of IPSS, improved peak flow rate and QoL [9]. In addition, the overall adverse events and complications such as acute urinary retention, postembolization syndrome and treatment failure were higher in the PAE group than in the TURP group. Nevertheless, Petrillo et al. reported a serious adverse event rate of less than 1% [7]. PAE is increasingly being used to treat BPH in patients with LUTS. However, Abt et al. reported some disadvantages of PAE in a 2 year, single-center, open-label, randomized trial [10]. Although the complications of PAE are less than TURP, the treatment effect is inferior. Furthermore, the late development of prostate cancer after PAE was less reported. Here, we report a patient with prostate cancer who had undergone PAE for the prostate 2 years before and discuss the potential mechanisms.

To the best of our knowledge, this is the first case presentation regarding prostate cancer occurring two years after PAE of the prostate. The PSA level increased rapidly after PAE, and the pathology report showed high-risk prostate cancer. The patient had not been well evaluated by a urologist before PAE was performed by a cardiologist. Although his PSA level was abnormal, he did not receive a complete cancer survey before undergoing PAE. Kuang et al. reported no significant change in PSA after PAE [8]. In our patient, his PSA level initially decreased after PAE. However, an abrupt increase was observed 3 months after PAE. He did not visit a urologist until the symptoms had worsened. Therefore, we recommend that patients should be well evaluated before and after PAE. There is a trend of minimally invasive treatment for BPH such as laser ablation, photoselective vaporization and PAE. There is not enough specimen for pathology interpretation. Therefore, the follow-up PSA value after the procedure is important for the detection of prostate cancer. However, the evolution or natural history of the serum PSA value after PAE has yet to be demonstrated in the literature. Shingleton et al. studied 120 patients with BPH who underwent TURP, photoselective vaporization and laser ablation after one and two years to determine the PSA change [11]. The PSA value decreased after the procedure, except in three patients who were diagnosed with prostate cancer due to rising PSA. The detection of non-reset PSA is an important clue of cancer formation. The follow-up protocol for patients who underwent PAE should be adapted to other minimal focal treatments of the prostate, such as Furusawa et al. who followed patients who underwent photoselective vaporization for BPH [12]. They claimed that patients should check the PSA value at 3 and 12 months, and each year after the procedure.

In addition, the incidence of prostate cancer steadily increases with age [13]. Anderson et al. analyzed a group of cancer patients with an average age of 80.5 years, and found that 11.7% of the male patients had prostate cancer [14]. In addition, an autopsy study of males aged over 80 years revealed that 43% had occult prostate cancer [15]. As less invasive screening for prostate cancer is recommended for males aged over 80 years [16], we suggest that PSA should be repeatedly checked in patients after PAE. An international multidisciplinary (urologist and radiologist online questionnaires) consensus regarding follow-up focal therapy for localized prostate cancer suggested that PSA should be checked every 3 months in the first year and every 6 months in the next year [17]. We agree with this consensus and recommend checking PSA every 3 months in the first year after PAE for patients aged over 80. For any abnormal rapid increase in PSA, a biopsy should be performed to rule out the possibility of prostate cancer.

The Warburg effect has been proposed to explain the progression of cancer after an intervention [18]. This effect was first observed by Warburg, who reported enormous amounts of sugar uptake with aerobic glycolysis around tumor cells [19]. Although the mechanism of the Warburg effect remains unclear, it has been proposed to be essential for tumor growth. As tumor cells must compete for energy with the surrounding tissue, aerobic glycolysis increases rapidly to provide ATP [20]. The Warburg effect may provide a tumor microenvironment that facilitates tumor growth. Increased growth signaling due to the Warburg effect is also a possible mechanism for tumor proliferation [21].

Embolization causes ischemia/hypoxia of tissues, which is harmful for normal cells and cancer cells and results in apoptosis, autophagy, necrosis and necroptosis [22]. However, normal cells are more prone to die than cancer cells in hypoxic conditions. Cells undergo a variety of responses to ischemia, and ischemia may increase tumor cell growth via several signaling pathways [23]. Cancer cells grow in a hypoxic microenvironment via genetic alterations or adaption [24]. One response of cells to hypoxia is via hypoxia-inducible transcription factor 1 (HIF-1). HIF-1 activates the expression of vascular endothelial growth factor (VEGF), which then increases angiogenesis [25]. VEGF is also involved in tumorigenesis. Other reactions include cell proliferation, metabolism, apoptosis, migration and immortalization [26]. Therefore, hypoxia is a key regulator of tumor growth which involves a series of transcription factors. Most of the response pathways promote tumor growth.

A selective microenvironment is known to facilitate the growth and progression of tumor cells [27]. This raises the question of whether normal cells become malignant after embolization via genetic alterations. Blood supply to the prostate is reduced after arterial embolization, which induces hypoxia. Re-perfusion or re-oxygenation after hypoxia may increase the production of free radicals which are regarded to increase tumor cell metastasis and result in a poor prognosis [28].

Mahal et al. studied the relationship of cancer-specific mortality among PSA level and Gleason score in 328,904 men with prostate cancer from clinically T1 to T4. The results indicate that a low PSA level (<2.5 ng/dL) with a high grade Gleason score (>8) significantly predict the poor prognosis of high cancer-specific mortality [29]. They hypothesized that a low PSA level with high pathological grade may indicate an aggressive and very poorly differentiated or anaplastic low PSA-producing tumors in a prostate cancer patient. Our patient has a high Gleason score and relatively low PSA level with boney metastasis diagnosed initially. However, he is under antiandrogen therapy with a relatively low PSA level. However, the follow-up time is too short to make a conclusion. Further observation of his clinical outcomes will confirm this issue.

In conclusion, this is the first report of a patient with BPH who developed prostate cancer within 2 years after PAE. The Warburg effect, hypoxia and older age are possible explanations. We suggest that PSA should be regularly checked, and DRE performed for patients who have been treated with PAE for voiding symptoms. It is still possible to develop prostate cancer even though the blood supply has been embolized. A rapid increase in PSA after PAE should be further evaluated for the possibility of prostate cancer.

## Figures and Tables

**Figure 1:**
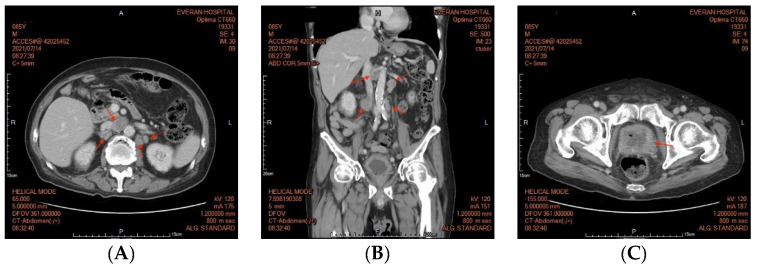
(**A**) Abdominal computed tomography (CT) revealed multiple lymph node enlargement at the paraaortic area (arrowhead). (**B**) Sagittal view of the abdomen CT. (**C**) Abdominal CT of the pelvis revealed a prostate gland with heterogenous density.

**Figure 2:**
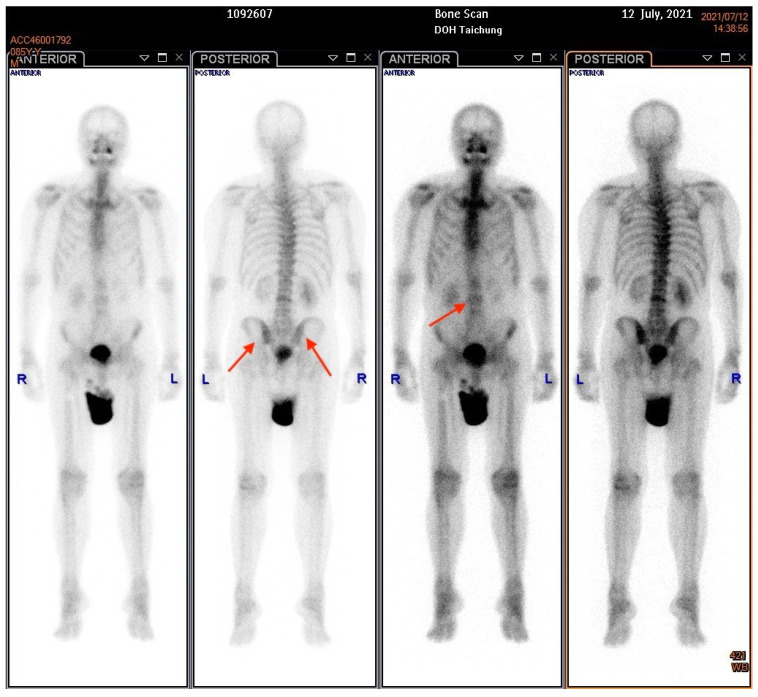
A bone scan demonstrated multiple boney metastases over the l-spine and pelvic wing. The arrows indicate the boney metastasis sites.

## Data Availability

All of the data are available upon request to the corresponding author.
